# *Osiris* gene family defines the cuticle nanopatterns of *Drosophila*

**DOI:** 10.1093/genetics/iyae065

**Published:** 2024-04-23

**Authors:** Zhengkuan Sun, Sachi Inagaki, Keita Miyoshi, Kuniaki Saito, Shigeo Hayashi

**Affiliations:** Laboratory for Morphogenetic Signaling, RIKEN Center for Biosystems Dynamics Research, 2-2-3 Minatojima-minamimachi, Chuo-ku, Kobe, Hyogo 650-0047, Japan; Department of Biology, Kobe University Graduate School of Science, 1-1 Rokkodai-cho, Nada-ku, Kobe, Hyogo 657-8051, Japan; Laboratory for Morphogenetic Signaling, RIKEN Center for Biosystems Dynamics Research, 2-2-3 Minatojima-minamimachi, Chuo-ku, Kobe, Hyogo 650-0047, Japan; Department of Chromosome Science, National Institute of Genetics, Research Organization of Information and Systems (ROIS), 1111 Yata, Mishima, Shizuoka 411-8540, Japan; Graduate Institute for Advanced Studies, SOKENDAI, 1111 Yata, Mishima, Shizuoka 411-8540, Japan; Department of Chromosome Science, National Institute of Genetics, Research Organization of Information and Systems (ROIS), 1111 Yata, Mishima, Shizuoka 411-8540, Japan; Graduate Institute for Advanced Studies, SOKENDAI, 1111 Yata, Mishima, Shizuoka 411-8540, Japan; Laboratory for Morphogenetic Signaling, RIKEN Center for Biosystems Dynamics Research, 2-2-3 Minatojima-minamimachi, Chuo-ku, Kobe, Hyogo 650-0047, Japan; Department of Biology, Kobe University Graduate School of Science, 1-1 Rokkodai-cho, Nada-ku, Kobe, Hyogo 657-8051, Japan

**Keywords:** insect, cuticle, nanostructure, sensory organ, triplo-lethal locus

## Abstract

Nanostructures of pores and protrusions in the insect cuticle modify molecular permeability and surface wetting and help insects sense various environmental cues. However, the cellular mechanisms that modify cuticle nanostructures are poorly understood. Here, we elucidate how insect-specific *Osiris* family genes are expressed in various cuticle-secreting cells in the *Drosophila* head during the early stages of cuticle secretion and cover nearly the entire surface of the head epidermis. Furthermore, we demonstrate how each sense organ cell with various cuticular nanostructures expressed a unique combination of *Osiris* genes. *Osiris* gene mutations cause various cuticle defects in the corneal nipples and pores of the chemosensory sensilla. Thus, our study emphasizes on the importance of *Osiris* genes for elucidating cuticle nanopatterning in insects.

## Introduction

Different forms of extracellular materials cover the body surface of nearly every higher animal and plant, such as stratum corneum, cell wall, or cuticle, which protects the fragile internal body environments from various lethal external environment factors like toxic chemicals, genotoxic radiation, and predators. These extracellular matrices are denucleated remnants of keratinocytes in the vertebrate epidermis or cellulose-based plant cell walls.

In insects, cuticles are multilayered structures comprising chitin-rich procuticles covered by proteins and lipid-rich epicuticles secreted sequentially by epidermal cells from the exterior to the interior of the organism. (Wigglesworth 1948). The cuticles harden to form protective shells that serve as exoskeletons. In addition, the cuticles of sensory organs serve as a window for receiving environmental cues, such as light, chemicals, and mechanical stimuli (Stocker 1994). The insect sensillum comprises hair (bristle) and socket cuticles, secreted by the trichogen and tormogen cells, respectively (Shanbhag *et al*. 1999). Sensory neurons innervate hair cell cuticles and are associated with the glia and sheath cells. All the cells in each sensillum descend from a single sensory precursor cell that is uniquely fated for a specific sensory lineage (Hartenstein and Posakony 1989).

The cuticles of the sensory organs adopt specific nanostructures to optimize the reception of each type of environmental signal. The cuticle of the mechanosensory bristles is supported by longitudinal pillar-like bulges that enhance the mechanical strength of the bristle, such that its deflection caused by any sensitive mechanical contact or air vibration is effectively transmitted to mechanosensory neurons innervating the base of the bristle. Cuticles of olfactory bristles contain multiple pores; the nanopore of 30–100 nm in diameter serves as a molecular filter, allowing the entry of airborne olfactory molecules of up to a few nanometers and preventing the entry of larger particles of dust and viruses (Steinbrecht 1997; Hunger and Steinbrecht 1998; Shanbhag *et al*. 1999). Regarding the gustatory sensillum, a single tip pore is used to incorporate water-soluble taste molecules into food (Shanbhag *et al*. 2001). The corneal nipples are equally spaced at ∼200 nm high protrusions covering the corneal lens (Kryuchkov *et al*. 2011). It deflects water droplets, self-cleans the corneal surface, and decreases light reflection. While industrial fabrications that mimic these surface structures attract attention from engineers (Bhushan 2009), the biological processes of cuticle nanostructure formation and their underlying genetic mechanisms have not been elucidated.

A recent study of the *Drosophila* olfactory organ with cuticle nanopores helped resolve this problem. We previously reported that *Osiris23*/*gore-tex* (*Osi23*/*gox*) is expressed in the olfactory hair cell (trichogen) on day 2 of pupal development, when the outermost layer of the epicuticle (envelope) is secreted (Ando *et al*. 2019). The nanopores were derived from the indentation of the envelope layer. In *Osi23*/*gox* mutants, the envelope indentation is flattened, nanopores are lost, and the mutant insects exhibit a reduced olfactory response. Since *Osi23*/*gox* mutant adults are viable and fertile with a normal external shape at the macroscopic level, this gene functions specifically in the nanolevel patterning of the cuticles.

*Osi23*/*gox* belongs to the *Osiris* gene family of 25 homologous genes in the *Drosophila* genome (Flybase, Gramates *et al*. 2022). Twenty-two *Drosophila Osiris* genes are clustered in the chromosomal region 83E, corresponding to the triple-lethal region, which shows unusual dosage sensitivity; either 1 or 3 copies of the region are lethal (Dorer *et al*. 2003; Lindsley *et al*. 1972). *Osi* gene family is present in many insect genomes, spanning the basal groups of mayflies and silverfish to highly evolved dipterans. However, no *Osi* homologs were found in the genomes of the basal hexapods (bristletails, Archaeognatha), crustaceans, or other arthropods. Molecular phylogenetic analysis revealed that specific classes of *Osi* genes from different insects clustered together, suggesting that the *Osi* gene was acquired in the early stages of insect evolution, which rapidly increased in number and then diverged (Shah *et al*. 2012). Although a few studies have addressed the function of specific *Osi* genes (Scholl *et al*. 2018, 2023; Smith *et al*. 2018; Scalzotto *et al*. 2022), a comprehensive analysis of the expression and genetic mechanisms of the *Osi* gene family in *Drosophila* or other insects is lacking.

In this study, we performed gene expression analysis of all *Osi* genes in the *Drosophila* head during the pupal stage. The results showed that in the early stages of adult cuticle deposition, *Osi* gene transcripts were found exclusively in specific cuticle-secreting cells in patterns unique to each *Osi* gene. Collectively, most adult cuticles are secreted by cells expressing specific combinations of *Osi* genes. Furthermore, systematic gene knockout experiments showed varying degrees of requirement, from haploinsufficiency for viability to no apparent requirement for adult viability and fertility. Four adult viable *Osi* mutants showed specific defects in the olfactory nanopores, gustatory tip pores, and corneal nipples in the eye. These results indicate that *Osi* is a candidate gene family that plays an essential role in cuticle nanostructure patterning in insects.

## Materials and methods

Key resources used in this research are listed in Supplementary Table 1.

### Experimental models

All *Drosophila* strains were cultured in a standard yeast–cornmeal media at 25°C. Fly pupae in the white prepupal stage were selected and staged.

### RNA probe preparation

Antisense RNA probes for each *Osiris* gene were amplified from DNA templates and PCR amplified from the genomic DNA of *the w* strain or *Osiris* cDNA clones. Digoxigenin- or biotin-labeled probes were synthesized using a labeling kit (Roche, Basel, Switzerland). The template DNA for the *Osi10b* RNA antisense probe was amplified using a primer set (forward: GTGGCGCGTCGTTTTACTAC, reverse: TAATACGACTCACTATAGGGCTTGATCGAGGCCCAGCTC). Primer sequences for other genes and the probe preparation method have been described previously (Ando *et al*. 2019).

### Fixation of *Drosophila* pupa for fluorescence in situ hybridization

Pupal heads for fluorescence in situ hybridization (FISH) experiments were prepared from pupae at 42 h after puparium formation (APF). The pupae were removed from the pupal case and poked with forceps at the posterior abdomen to increase the permeability of the fixative. The pupae were then transferred into ∼250 μL of 4% paraformaldehyde in phosphate-buffered saline (PBS) and incubated for 16 h at 4°C. The pupal cuticle was then removed using fine forceps. The pupal heads (with legs and wings) were collected and rinsed with PBS-Tween (PBST, 0.1% Tween-20 in 1× PBS). The fixed pupal heads were dehydrated by washing with 25, 50, 80, and 100% ethanol for at least 10 min each. After 1 more wash with 100% ethanol, they were stored at −20°C. The incubations and rinsing of the dehydration steps were performed at room temperature with 500 μL of each solution. All rinsing steps were performed for at least 5 min.

### Single-color FISH

This procedure was modified from a previously described protocol (Inagaki *et al*. 2005). Fixed and dehydrated pupal heads were incubated in a 2-mL microcentrifuge tube with 1 mL of a 1:1 mixture of xylene and ethanol for 60 min. The heads were rinsed twice in 100% ethanol and rehydrated using a graded series of ethanol solutions (80, 50, and 25% ethanol) and water. Rehydrated pupal heads were incubated in a 4:1 acetone/water solution at −20°C for 10 min. Subsequently, the heads were rinsed twice with PBST and refixed in 4% paraformaldehyde in PBS for 20 min and then rinsed in PBST 5 times. The pupal heads were prehybridized using prehybridization solution (50% formamide, 5× saline–sodium citrate (SSC) buffer, 100 μg/mL heparin, 0.1% Tween-20, 100 μg/mL yeast RNA, and 10 mM dithiothreitol) at 61.7°C for 60 min. The prehybridization solution was replaced with the hybridization solution (50% formamide, 5× SSC, 100 μg/mL heparin, 0.1% Tween-20, 100 μg/mL yeast RNA, 10 mM dithiothreitol, and 10% dextran sulfate) with a final concentration of 0.6 ng/μL digoxigenin-labeled *Osiris* RNA probe. Heads were hybridized overnight at 61.7°C in a rocking incubator. The pupal heads were washed in a series of wash solutions (50% formamide in PBST mixed with 5×, 4×, 3×, 2×, and 1× SSC). Each wash was repeated 3 times for 5 min at 61.7°C.

The heads were then rinsed 5 times for 5 min in PBST and incubated in the Blocking Reagent (Roche, 1:5,000 dilution) for 60 min. Then, the heads were incubated in the mixture of Anti-Digoxigenin-POD (Roche, 1:500 dilution), anti-Futsch (DSHB, Iowa City, IA, USA, 1:10 dilution), and phosphotyrosine (Cell Signaling Technology, Danvers, MA, USA, 1:200 dilution) in PBST overnight at 4°C. The heads were rinsed 5 times in PBST at room temperature (approximately 26°C) and then incubated in 50× diluted Cy3 Tyramide Reagent (PerkinElmer Life Science Inc., Shelton, CT, USA, 1:50 dilution) in an amplification dilution buffer for 90 min at room temperature. The reaction was terminated by rinsing with the Blocking Reagent 3 times for 10 min each. Subsequently, the samples were incubated for 90 min in anti-rabbit Alexa Fluor 488 (Invitrogen, Waltham, MA, USA, 1:500 dilution) to detect phosphotyrosine and anti-mouse Alexa Fluor 633 (Invitrogen, 1:500 dilution) to detect Futsch. Finally, the samples were rinsed in PBST 3 times and mounted in an Antifade Mounting Medium with DAPI (VECTASHIELD, Vector Laboratories, Inc., Newark, CA, USA).

### Two-color RNA FISH

After prehybridization and blocking, pupal heads were incubated overnight in the hybridization solution comprising 0.6 ng/μL each digoxigenin- and biotin-labeled RNA probe. After washing and blocking the hybridization solution, the samples were incubated overnight with streptavidin–peroxidase conjugate (Roche, 1:500 dilution) and washed. A Tyramide amplification reaction (Cy3) was performed (see *Single-color FISH*). After the reaction, the sample was treated with 0.01 M HCl for 10 min to inactivate the peroxidase (Lécuyer *et al*. 2008). The samples were rinsed with PBST, and blocking was repeated. Then, anti-digoxigenin-POD and anti-Futsch (or phosphotyrosine) were added simultaneously and incubated overnight at 4°C. The immune reaction was terminated by washing with PBST 3 times. Another Tyramide amplification reaction (FITC) was performed for 90 min (PerkinElmer Life Science Inc., 1:50 dilution in amplification buffer). After the TSA reaction, the samples were washed and incubated for 90 min with 1:500 anti-mouse Alexa Fluor 633 to detect Futsch (or 1:500 anti-rabbit Alexa Fluor 633 to detect phosphotyrosine). Finally, the samples were washed 3 times with PBST and mounted in an Antifade Mounting Medium with DAPI.

### Imaginal disk staining

Third-instar larvae of the *Osi17* mutant mosaic experiment were dissected, and the wing, haltere, and hind leg disks were fixed in 4% paraformaldehyde in PBS for 40 min at room temperature. Tissues were blocked with 0.1% bovine serum albumin (BSA) in PBST (0.1% Triton-X in PBS) 3 times for 10 min each. The disks were incubated with 1:400 diluted Alexa Fluor 568 Phalloidin in PBST containing BSA for 1 h. Finally, the disks were washed 3 times and mounted using an Antifade Mounting Medium with DAPI.

### Sample preparation for field emission scanning electron microscopy

The adult *Drosophila* heads were dissected in PBS and then rinsed with 0.1 M cacodylate buffer 3 times, over 5 min each, and incubated in fixation buffer 1 (2% paraformaldehyde, 2.5% glutaraldehyde, and 0.1 M cacodylate buffer) at 4°C overnight. The samples were rinsed with 0.1 M cacodylate buffer 3 times at room temperature for over 5 min each. The samples were then incubated in fixation buffer 2 (1% osmium tetroxide and 0.1 M cacodylate buffer) on ice for 120 min under dark conditions. The adult heads were further rinsed in water 3 times on ice under dark conditions and subsequently dehydrated in an ethanol gradient (25, 50, 75, 80, 90, 95, 99.5, and 100%) for 10 min each at room temperature. Finally, 100% ethanol was dehydrated using a molecular sieve. The samples were dried overnight under a vacuum. After dehydration, the heads were mounted on a double-sided carbon tape on a brass pedestal and coated with osmium tetroxide to a thickness of approximately 13 nm using an osmium coater (Tennant 20, Meiwafosis Co. Ltd., Tokyo, Japan).

### Image acquisition

Fluorescent images were captured using a confocal microscope (FV1000, Olympus, Tokyo, Japan) under a 10× objective lens (NA 0.40) for whole pupal head scans and a 60× water immersion objective lens (NA 1.20) for high-resolution images of the antenna, palps, distiproboscis, and eyes. For higher resolution images, 0.54 μm z-stacks were taken. All image data were analyzed using Fiji ImageJ software (Schindelin *et al*. 2012).

External views of the adult flies were observed using a field emission scanning electron microscope (FE-SEM; JSM-IT700HR, JEOL, Tokyo, Japan). A Helium Ion Microscope (ORION Plus, Carl Zeiss, Wetzlar, Germany; installed at the nanoprocessing facility at AIST Tsukuba, Japan) was used during the early screening stage.

### Image processing

To map *Osi23*/*gox* expression on the curved surface of the third antennal segment (An3), we used the ImageJ plugin “SheetMeshProjection” (Wada and Hayashi 2020). This tool allows the conversion of curved surfaces of objects into 3D stacks of cut open flat views. The correlation between the olfactory organs, identified by phosphotyrosine staining, and strong and weak *Osi23*/*gox* expression was confirmed by moving through the stacks.

### Genome editing

Gene knockout strains were produced using the transgenic guide RNA and Cas9 methods (Kondo and Ueda 2013). Multiple alleles were identified for each gene. Complementation tests with a deficiency chromosome were not possible due to haploinsufficiency of the locus (Lindsley *et al*. 1972). Lethality was judged when all alleles were homozygous and lethal (Table 1; Supplementary Table 3). Knockout strains of *Osi6* were not recovered after the trial with 2 different guide RNAs, possibly due to the haploinsufficiency of this gene.

**Table 1.** Osi expression patterns.

	Trichogen			Tormogen	Lens	Epi	Pseudotrachea	Arista	Phenotype
***Osi***	Olf	Mech	Gustatory						
**1**					+			+	
**3**				+		+			
**4**	+	+	+	+	+		+	+	cn
**5**	+								
**6**					+		+		
**7**				+	+	+	+		
**8**		+	+					+	
**9**					+	+			cn
**11**		+	+					+	gb
**12**				+		+	+	+	
**13**	+								
**16**	+								
**21**		+	+						
**22**						+		+	
**23**	+								sb, st
**24**	+	+							

Olf, olfactory organ; Mech, mechanosensory organ; Epi, epidermis; cn, corneal nipple; gb, gustatory bristle; sb, sensilla basiconica; st, sensilla trichordia.

## Results

### Unique combination of *Osiris* gene expression prefigures morphogenesis of specific cuticle structures

**Fig. 1.**
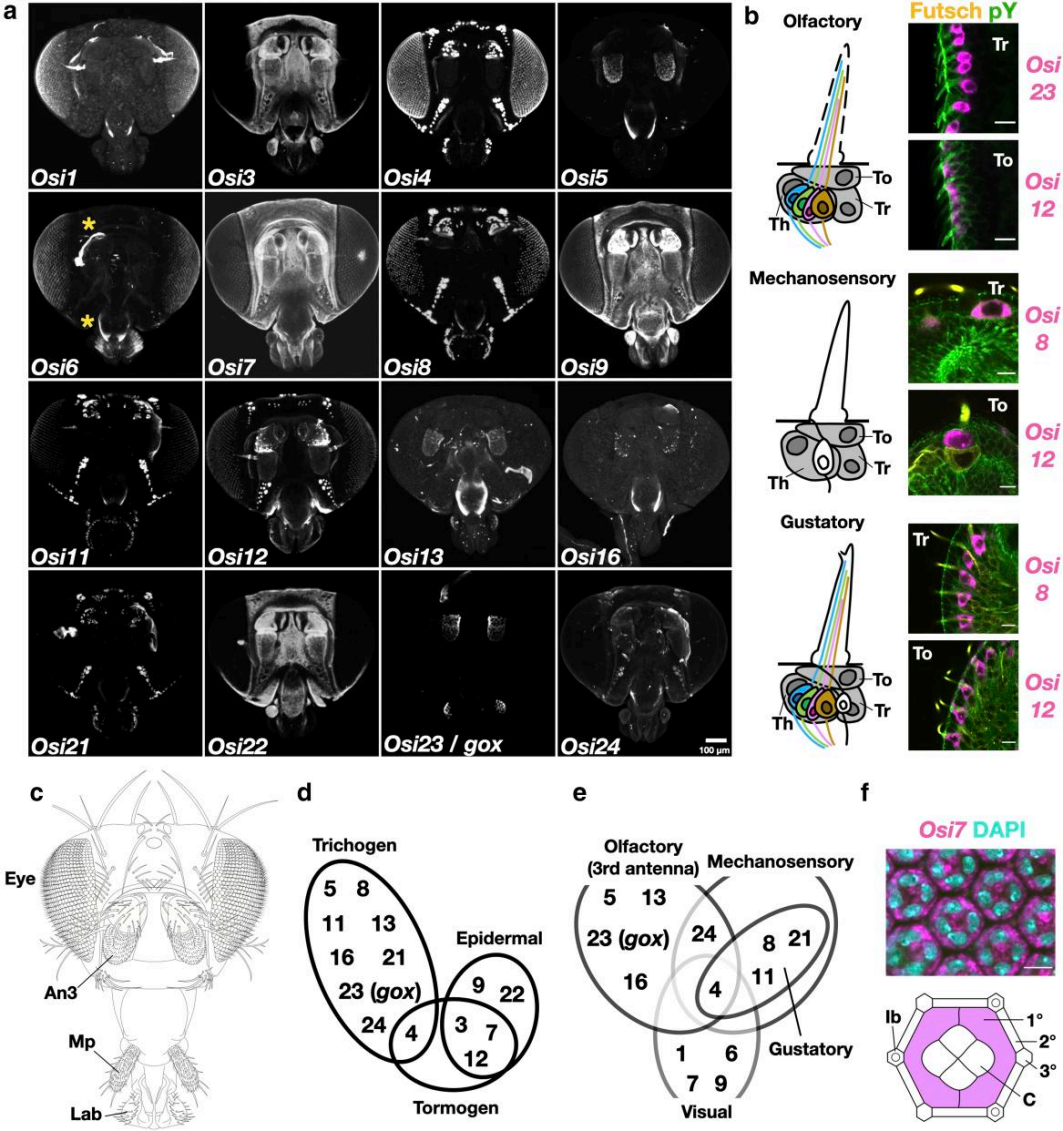
Expression patterns of *Osi* genes in the pupal head. a) mRNA expression of 16 *Osi* genes in 42 h APF pupal heads. Asterisk (*) indicates a nonspecific signal to the pupal cuticle remnants. b) Schematics of 3 sensory bristle types and examples of pupal *Osi* gene expressions in To (tormogen cell) and Tr (trichogen cell). Th, thecogen cell. mRNA (magenta), phosphotyrosine (green, cell junction), and Futsch (yellow, bristle shaft and neuron). c) A schematic of *Drosophila* adult head. An3, third antennal segment; Mp, maxillary palp; Lab, labellum. d) Summary of *Osi* gene expressions in 3 types of cuticle-secreting cells. e) Summary of *Osi* gene expressions in different sensory organ types. Note that *Osi* expressions in the gustatory organ are a subset of expressions in the mechanosensory organ. f) An example of *Osi* expression in the compound eye. *Osi7* mRNAs were detected in primary pigment cells (1°) and cone cells (C). 2° and 3°, secondary and tertiary pigment cells. mRNA (magenta) and DNA (cyan). The scale bar is 10 µm unless otherwise indicated.

The expression patterns of *the Osiris* genes in *Drosophila* embryo have been previously described (Ando *et al*. 2019). We sought to study the tissue expression patterns of *Osiris* genes in the head of pupae at 42–44 h APF (Fig. 1c), when the levels of *Osiris* gene transcripts peaked at the pupal stage (Brown *et al*. 2014; Sobala and Adler 2016; Larkin *et al*. 2021), and the expression of *Osi23*/*gox* was detected in the olfactory hair cells (Ando *et al*. 2019). *Osi23*/*gox* starts expression when the envelope layer of the cuticle is assembled before production of chitin and other components of the procuticle (Sobala and Adler 2016; Ando *et al*. 2019). We reasoned that if other *Osiris* genes play a role analogous to *Osi23*/*gox* in the nanopatterning of the cuticle through modulation of the envelope shape, they would be expressed at this stage of envelope formation.

**Fig. 2.**
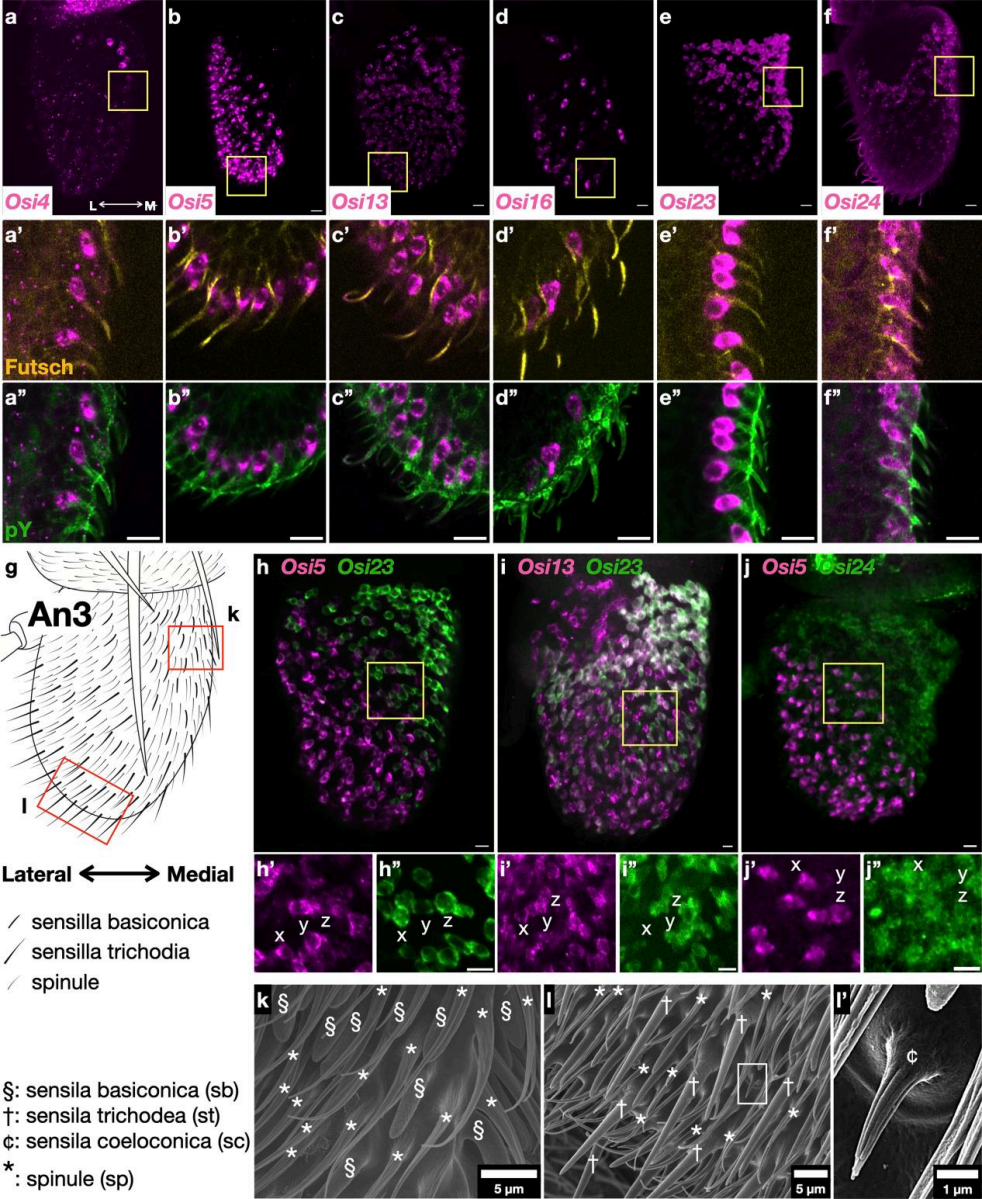
*Osi* gene expressions in olfactory organs. a–f) Overview of *Osi* gene expressed in the third antennal segment (An3). L, lateral; M, medial. a’–f’ and a”–f”) Magnified views of yellow boxes in a–f). Phosphotyrosine (green) and Futsch (yellow). g) Schematic of An3. sb and st are enriched in the medial top and lateral bottom regions, respectively. The SEM images of each region are presented in k) and l). h–j) Two-color FISH images of 3 pairs of *Osi* mRNA expression. h’–j’ and h”–j”) High magnification views of the yellow boxes. Note that *Osi23* expression overlaps significantly with *Osi13* i), but is distinct from that of *Osi5* h). *Osi24* expression differs from *Osi5* j). k) SEM image of the top medial region enriched with sb. l) Lateral bottom region enriched with st. l’) Enlarged view of the sc (white box in l).

**Fig. 3.**
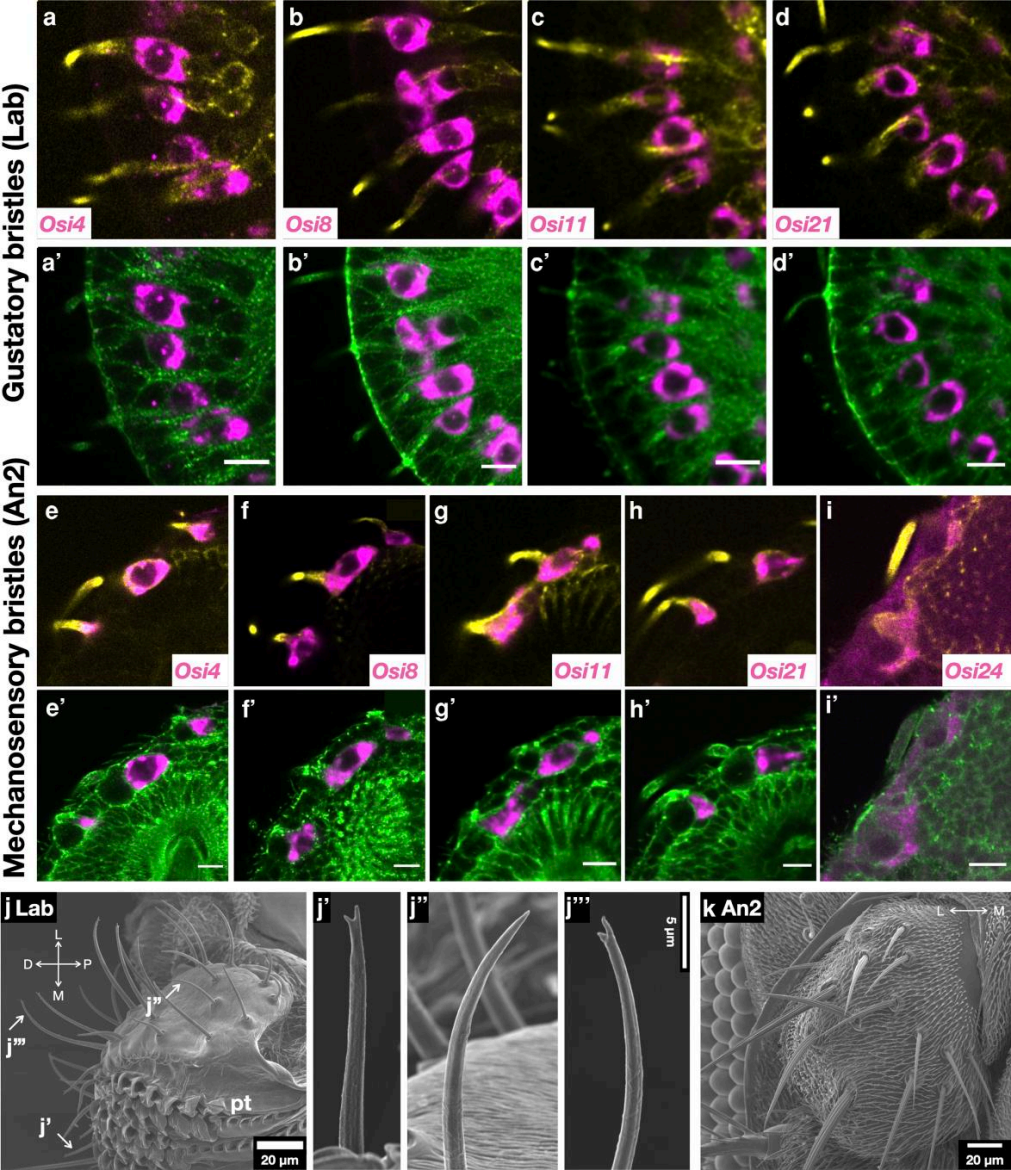
*Osi* gene expressions in gustatory and mechanosensory organs. a–i) Expressions of *Osi* genes expressed in trichogen colabeled with Futsch. a’–i’) *Osi* expressions colabeled with phosphotyrosine. j) Scanning electron microscopy image of the labellum. j’) Small taste bristle. j”) Intermediate taste bristle. j”’) Large taste bristles. D, distal; P, proximal; L, lateral; M, medial; pt, pseudotrachea. k) SEM images of the An2 with mechanosensory bristles.

**Fig. 4.**
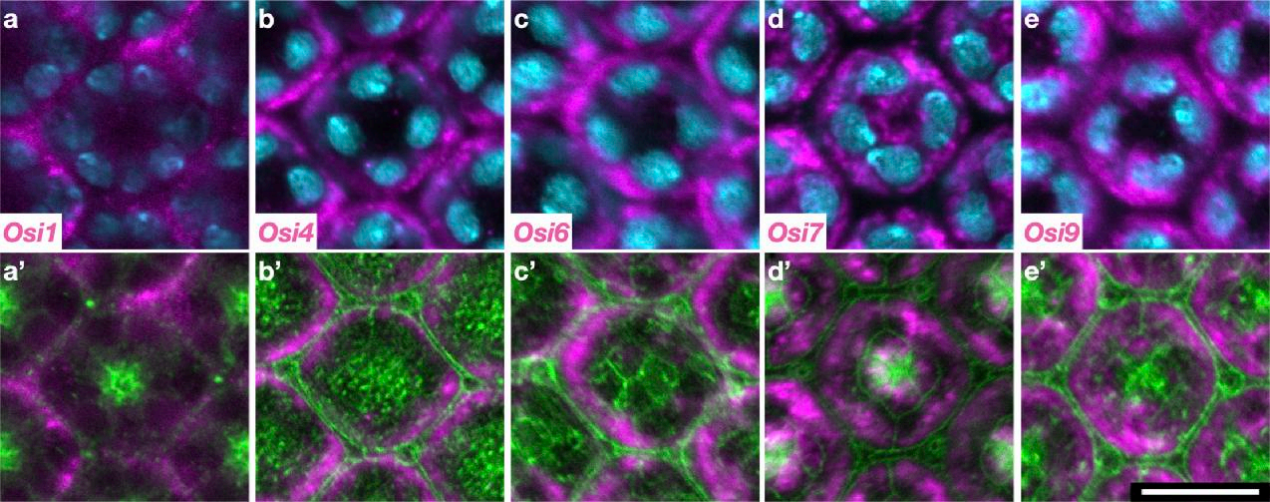
*Osi* gene expressions in the compound eye. a–e) *Osi* expressions (magenta) with nuclei (cyan). a’–e’) *Osi* expression with phosphotyrosine (green). Approximate depth from the apical surface: 4.86 μm (*Osi1*), 1.62 μm (*Osi4*), 1.62 μm (*Osi6*), 2.16 μm (*Osi7*), and 1.62 μm (*Osi9*).

FISH was performed on the whole head using 25 probes for each *Osi* RNA (*Osi1*–*Osi24*; *Osi10* was reannotated as *Osi10a* and *Osi10b*, colored magenta, Fig. 1a; Supplementary Figs. 1 and 2). The samples were costained with anti-phosphotyrosine (green) and anti-Futsch (yellow) antibodies to reveal the cell outline, bristle shaft cells (trichogen), and neurons, respectively, and the nuclei were labeled with DAPI (cyan, Supplementary Figs. 1 and 2). Based on the low magnification views, *Osiris* expression patterns were classified into 3 categories (Fig. 1a; Supplementary Figs. 2 and 3.1–3.5 and Table 1). The first group of genes (*3*, *7*, *9*, and *22*) was mainly expressed in the epidermal cells, and the second group (*1*, *4*, *5*, *6*, *8*, *11*, *12*, *13*, *16*, *21*, *23*, and *24*) was expressed in various sensory organs, including the eye, antenna, maxillary palp (Mp), and proboscis (Fig. 1a, b, and f). The third group of genes (*2*, *10a*, *10b*, *14*, *15*, *17*, *18*, *19*, and *20*; Supplementary Fig. 2) was not expressed at a detectable level in the head at this stage. We noted that our FISH assay was sensitive enough to detect robust sensory expression of *Osi16* and *Osi23*/*gox* RNAs that were classified as “low” expressed genes [5 fragments per kb of exon per million mapped reads (FPKM)] in the modENCODE temporal gene expression database (Graveley *et al*. 2011). Expression patterns of 16 *Osi* genes were grouped into 3 cuticle-secreting cell types (epidermis, trichogen, and tormogen, Fig. 1d) and sensory organ types (olfactory, mechanosensory, gustatory, and visual systems, Fig. 1e). The detailed expression of each *Osi* gene is presented in Figs. 1–4 and Supplementary Fig. 3.

### Olfactory sensillum

The expression of 6 *Osi* genes in olfactory organs is shown in Fig. 2a–f. The olfactory organs are categorized as sensilla basiconica (sb), sensilla trichordia (st), and sensilla coeloconica (sc), each with multiple cuticular nanopores (Shanbhag *et al*. 1999; Fig. 2g, k, l, and l’). These sensilla are further classified based on their size, olfactory receptor expression, and responses to specific chemicals (Chai *et al*. 2019). All 3 types of olfactory sensilla were present in An3, and only sb was present in the Mp. *Osi13*, *Osi23*/*gox*, and *Osi24* were detected in trichogen cells of Mp in patterns resembling the distribution of HA-Gox driven by the *Osi23*/*gox* promoter (Figs. 1b and 2; Supplementary Figs. 3-4 and 3-5; Ando *et al*. 2019), suggesting that these genes were expressed in sb. *Osi5* was abundantly expressed in An3 in a pattern complementary to the sb location labeled by strong *Osi23* (Fig. 2h; Supplementary Fig. 3-2). Based on its similarity to the st distribution in adult An3, *Osi5* is likely to be expressed in st. *Osi13* and *Osi23* were expressed mainly in overlapping patterns in An3 (Fig. 2i) and Mp (Supplementary Fig. 4). *Osi24* expression partially overlapped with that of *Osi5* (Fig. 2j). *Osi16* was expressed in a scattered pattern in An3 cells (Fig. 2d; Supplementary Fig. 3-4). It is possible that *Osi16* is also expressed in sc. The precise mapping of *Osi*-expressing cells requires colabeling with a marker for olfactory receptor genes assigned to specific sensilla types (reviewed in Vosshall and Stocker 2007).

### Mechanosensory and gustatory sensillum

Four *Osiris* genes (*Osi4*, *Osi8*, *Osi11*, and *Osi21*) were detected in all the trichogen cells of the gustatory bristles of the proboscis (Figs. 3a–d and 1b and e; Supplementary Figs. 3-1, 3-3, and 3-5). Five *Osiris* genes (*Osi4*, *Osi8*, *Osi11*, *Osi21*, and *Osi24*) were expressed in all trichogen cells of the mechanosensory bristles (Figs. 3e–i and 1b; Supplementary Fig. 1). In addition, *Osi3* expression was detected in gustatory tormogen cells (Supplementary Fig. 3-1). Mechanosensory bristles transmit mechanical stimuli to the mechanoresponsive nerve terminus, which is attached to one side of the bristle base. Their shape is characterized by prominent bulges running along the long axis of the bristle, which are prepatterned by actin bundles formed during the pupal stage (Fig. 3k;  Lees and Picken 1945; Tilney *et al*. 1995). Gustatory organs sense water-soluble chemicals using gustatory neurons inside a bristle. Gustatory bristle shafts have pillar-like bulges similar to mechanosensory bristles and a pore at each tip through which water and dissolved molecules reach the gustatory neurons inside the bristle (Figs. 1b and 3j). The gustatory bristles are innervated by mechanosensitive neurons (Jeong *et al*. 2016). The similarity in the bulge structures and the overlapping expression patterns of *Osi* genes imply that the gustatory and mechanosensory bristles share similar properties.

### *Osi* expression in the eye

*Osi1*, *Osi4*, *Osi6*, *Osi7*, and *Osi9* are expressed in the primary pigment cells of the compound eye (Figs. 1f and 4a–e; Supplementary Figs. 3-1, 3-2, and 3-3). *Osi7* was also expressed in the cone cells (Fig. 4d). These cells are involved in the secretion of the transparent lens cuticle. In addition, *Osi4* expression was detected in unidentified compound eye cells (Supplementary Fig. 3-1). However, it is unlikely that these cells are involved in lens secretion.

### Epidermis and arista

*Osi3*, *Osi7*, *Osi9*, and *Osi22* are expressed in most parts of the epidermis (Fig. 1a) and are covered by an epidermal protrusion called a spinule (sometimes called a trichome or hair). We noted that part of the central posterior part of the proboscis showed little or no expression of any *Osi* genes. Another area that lacked *Osi* expression was the horizontal strip above the antenna (Fig. 1c). This region is dorsal to the ptilinum and is folded inside the adult head.

*Osi1*, *Osi8*, *Osi11*, and *Osi12* are expressed in the distal part of the antenna (Supplementary Figs. 3-1, 3-3, and 3-4). *Osi1*, *Osi8*, and *Osi11* are expressed in the basal cylinder and further distal parts, with an enhanced expression on the dorsal side. *Osi12* showed a distinct ring pattern at the boundary between the basal cylinder and arista (Supplementary Fig. 3-4).

### Mutagenesis of *Osiris* gene family

Mutations in a subset of *Osiris* genes have been reported (Ando *et al*. 2019; Scalzotto *et al*. 2022; Scholl *et al*. 2023), but no comprehensive mutagenesis of the *Osiris* gene has been performed. Preliminary experiments on knockdown *Osi* genes in transgenic UAS-RNAi strains (Dietzl *et al*. 2007; Ni *et al*. 2008) yielded mixed results, where some RNAi constructs caused lethality, whereas others did not (Supplementary Table 2). We then performed a systematic knockout of all 25 *Osiris* genes using the CRISPR/Cas9 technique with transgenic guide RNA (Kondo and Ueda 2013; Table 1; Supplementary Table 3). Multiple small deletion alleles causing frameshift mutations in the open reading frame of each *Osiris* were recovered for 24 *Osiris* genes, among which 5 were lethal (*Osi6,*  *Osi7*, *Osi17*, *Osi20*, and *Osi24*) and 2 were semilethal (*Osi10a* and *Osi14*). However, we did not recover any protein-null mutation of *Osi6* after 3 attempts with different guide RNA constructs. One allele of lethal *Osi6* previously isolated was embryonic lethal and caused a strong cuticle defect (Ando *et al*. 2019). Heterozygous *Osi6* and *Osi7* stocks were weak and sluggish, indicating that a 1-dose reduction in these genes seriously affected the viability.

**Fig. 5.**
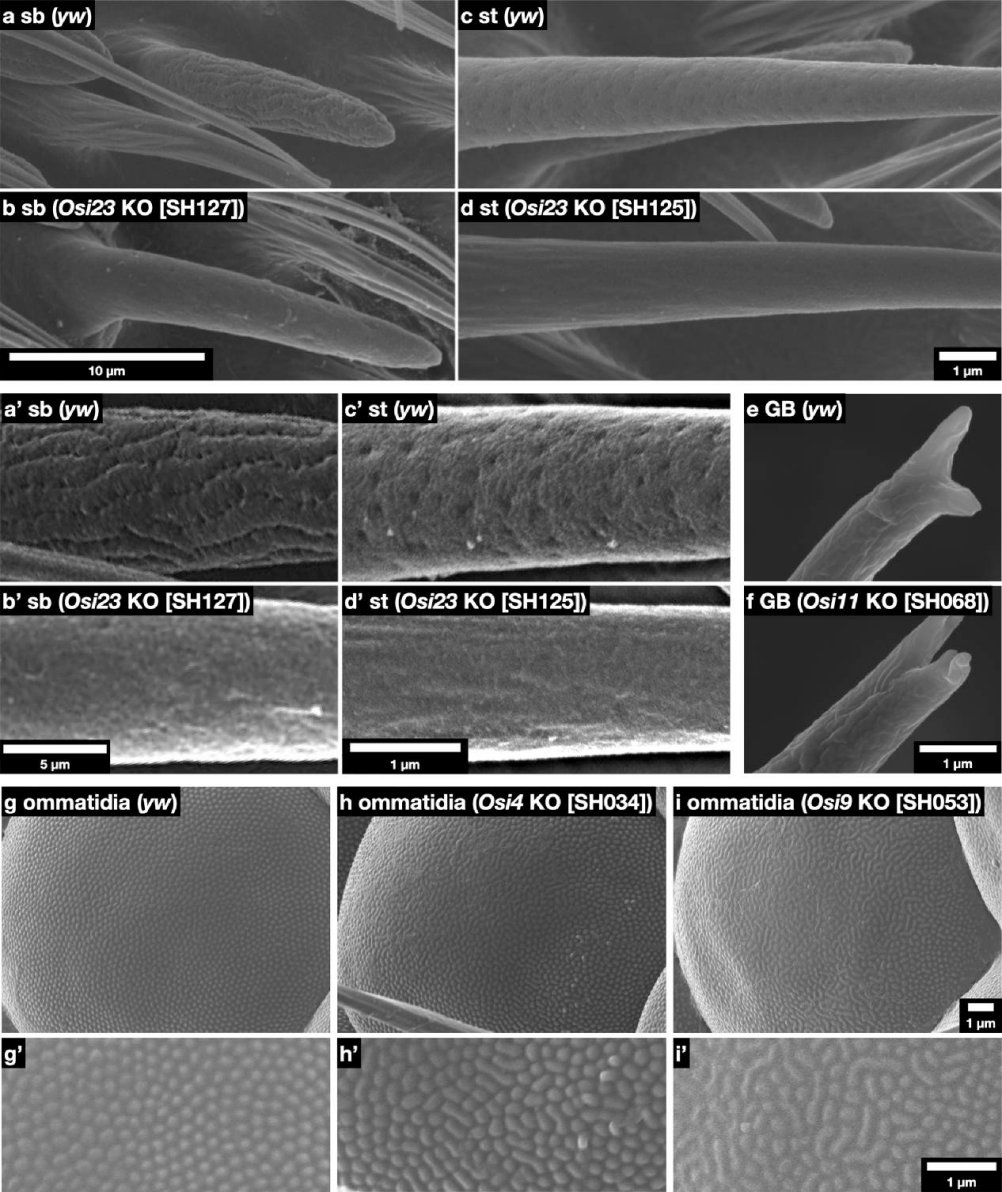
Impact of *Osi* gene mutations on cuticle nanostructure formation. a, a’, b, and b’) SEM images of sb in An3. c, c’, d, and d’) st in An3. Note the clear loss of nanopores in the sb and st. e, f) Tips with long gustatory hair. In *Osi11* KO, each tip was further bifurcated. g–i and g’–i’) Surface views of ommatidium. Individually separated nipple arrays in the control g) were laterally fused in *Osi4* and *Osi9* mutants.

The heads of viable adult mutants were examined by FE-SEM. The cuticle patterns of the antenna, compound eyes, and proboscis were observed at magnifications of up to 20,000× (Fig. 5). External morphology was observed in the antennae (olfactory organs in An3, mechanosensory organs in An2, and arista), Mp, Lab (labellum, gustatory organs, and pseudotrachea), and lenses of the compound eyes of multiple independent alleles of homozygous mutant adults of each gene (Table 1; Supplementary Table 3). Defects in the nanostructures were found in the olfactory organs, gustatory organs, and eye lenses, as described below.

### Phenotypes in the olfactory organs

**Fig. 6.**
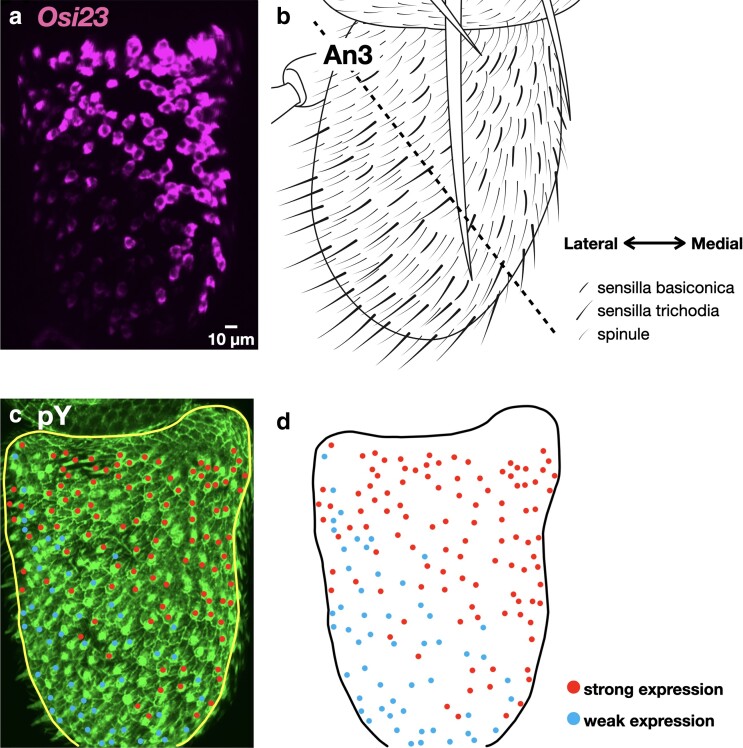
*Osi23* expression in An3. Cut open views of the top surface of An3. a) *Osi23* was strongly expressed in the top medial area enriched with sb and weakly expressed in the bottom lateral area enriched with st. b) Schematic representation of An3. Dotted line indicates approximate boundary of sb-enriched top medial region and st-enriched bottom lateral region. c, d) Anti-phosphotyrosine (pY) staining and the map of *Osi23* expression.

*Osi23*/*gox* mutants showed a loss of nanopores in the sb of the Mp, as previously reported (Ando *et al*. 2019). These mutations also caused a loss-of-nanopore phenotype in the sb of An3 (Fig. 5a, a’, b, and b’). Furthermore, we observed a loss of nanopores in the st of An3 (Fig. 5c, c’, d, and d’). We also examined the olfactory organ phenotypes in mutants *Osi4*, *Osi5*, *Osi13*, *Osi16*, and *Osi24* expressed in the bristles of olfactory organs in An3; however, no obvious phenotype was observed. To investigate the role of *Osi23*/*gox* in st morphogenesis, we reexamined its expression pattern in An3 at 42 h APF and found that many trichogen cells expressed *Osi23*/*gox* RNA at low levels in the ventrolateral region of An3, which is covered by st in adults (Fig. 6a–d; Supplementary Movie 1). These results imply that *Osi23*/*gox* contributes to nanopore formation in 2 types of olfactory hair cells, sb and st. The external appearance of sc was normal in *Osi23*/*gox* mutants.

### Phenotypes in the gustatory organ

Among the 4 *Osi* genes expressed in the gustatory organs (*Osi4*, *Osi8*, *Osi11*, and *Osi21*), mutants of *Osi11* showed a change in the morphology of the branched tips of long- and short-type gustatory hairs (Fig. 5e and f).

### Phenotypes in the lens

In control eyes, corneal nipples were ∼30 nm high protrusions spaced by ∼255 nm equally spaced on the surface of the lens (Kryuchkov *et al*. 2011). In *Osi4* and *Osi9* mutants, some corneal nipples are fused laterally to form a labyrinthine pattern (Fig. 5g, g’, h, h’, i, and i’). No specific defects were observed in *the Osi6* and *Osi7*.

### Phenotype of *Osi17* knockdown in the wing

Although *Osi17* was homozygous lethal, the RNAi-mediated knockdown of the *actin-Gal4* driver caused an eclosion defect with shrunken wings (Supplementary Fig. 5). Because *Osi17* is expressed in the embryonic tracheal system (Ando *et al*. 2019), we targeted the RNAi to the tracheal system using a tracheal driver. No wing defects were observed. Next, we selectively produced *Osi17* mutant clones using *the Ubx*-*flip* recombinase. Mutant flies reproduced the shrunken wing phenotype. Since recombination occurred in the wing pouch region but not in the trachea or adult muscle precursor cells associated with the wing disc, we concluded that *Osi17* function was required in the wing epithelium to produce a properly expanded wing.

## Discussion

In this study, we examined the expression patterns of *Osiris* family genes in the pupal heads at the earliest stage of adult cuticle formation. Of the 25 *Osi* genes, 16 were expressed in specific patterns in cuticle-secreting epidermal and sensory organ cells, 4 of which were required for specific cuticle nanostructures.

### *Osiris* functions in sensory bristle nanopatterns

Mutations in *Osi23*/*gox* caused defects in nanopore formation in both *sb* and *st*. This implied that a common *Osi23*/*gox-*dependent mechanism underlies nanopore formation in these sensilla. In contrast, tip pore formation in the gustatory bristles involves a different *Osi* gene, *Osi11*, suggesting that distinct mechanisms are involved in the formation of these 2 pore types. Consistent with this view, it was previously shown that the origin of the tip pore-type chemo-sensillum morphology might be traced back to a crustacean-like ancestor, while the nanopore-bearing olfactory sensillum was newly acquired in insects (Harzsch and Krieger 2018).

### *Osiris* functions in the corneal nipple nanopattern

Of the 5 *Osi* genes expressed in the lens cuticle-secreting cells, mutants of *Osi4* and *Osi9* showed defects in the pattern of nipple arrays. Lateral fusion of nipple arrays forms labyrinthine patterns that are reminiscent of the lens patterns observed in some *Drosophila* species and in *Drosophila melanogaster* mutants deficient in retinin and waxes that partly constitute to the nipple structures (Blagodatski *et al*. 2015; Kryuchkov *et al*. 2020). It is likely that *Osi4* and *Osi9* are components of the reaction–diffusion mechanism of corneal nipple array patterning (Turing 1990; Kryuchkov *et al*. 2020).

### *Osiris* gene functions in the epidermis

*Osi3*, *Osi7*, *Osi9*, and *Osi22* are strongly expressed in the epidermis. Although *Osi3*, *Osi9*, and *Osi22* are viable and did not cause obvious defects in the epidermal cuticle or trichome, it has been previously shown that embryonic lethal *Osi6* and *Osi7* mutants showed strong defects in larval cuticle formation (Ando *et al*. 2019). In addition, the lack of *Osi17* function causes wing expansion defects, likely due to the weakening of the epidermal cuticle. However, whether these defects reflect the function of the cuticle nanopatterns or general cuticle production remains unclear.

### Dynamic *Osiris* gene expression

For *Osi4*, we observed related but distinct expression patterns (Supplementary Fig. 3-1) in a batch of similarly staged pupae fixed at 42 h APF and processed together in a single tube. One head showed a high expression in the eye but not in the pseudotrachea. Another head exhibited weak eye expressions and prominent pseudotracheal expressions. It is possible that *Osi4* expression dynamically changes, and a slight difference in the developmental stage (less than ±0.5 h) causes a significant difference in the expression pattern. As described above, nanopore formation in st was sensitive to *the Osi23*/*gox* mutation, although its expression in st was low at 42 h APF. The stage of high *Osi23*/*gox* expression in st primordia may have been missed. Time-course analyses of the expression *of Osi23*/*gox* and other *Osi* genes are required to fully document the contributions of these genes to the complex morphogenesis of cuticle nanopatterns.

### Genetic requirement for *Osi* genes

Systematic knockout of *Osi* genes revealed variable requirements for each *Osi* gene in terms of organismal viability and cuticle nanopatterning. The requirement for *Osi6* and *Osi7* activities is especially high because the heterozygosity of either gene reduces animal fitness (Ando *et al*. 2019; this study). Five additional *Osi* genes were either lethal or semilethal. These results support the hypothesis that the combined effect of *Osi* genes accounts for the haploinsufficiency of the chromosomal locus 83D-E covering the complex of 22 *Osi* genes (Lindsley *et al*. 1972; Shah *et al*. 2012).

The 22 *Osi* genes were densely packed and sometimes overlapped in the ∼168 kb region of 83D-E. However, neighboring *Osi* genes are expressed in different patterns, arguing against a model of the coregulation of *Osi* gene at the chromosomal level.

Mutations in several *Osi* genes coexpressed in the bristles and epidermis did not cause notable changes in the cuticle, and genetic redundancy reported for *Osi9*, *Osi15*, and *Osi19* in tracheal function (Scholl *et al*. 2023) is likely the reason. The expression patterns reported in this study will provide a basis for future studies on multiple mutations in genes coexpressed in the same cell type, which can elucidate the full repertoire of *Osi* gene functions.

## Supplementary Material

iyae065_Supplementary_Data

## Data Availability

The resource origin and associated information are described in the key resource table. The *Osiris* knockout and guide RNA strains used in this study are available from the National Institute of Genetics (https://shigen.nig.ac.jp/fly/nigfly/). Original image stacks of FISH images of each *Osi* gene are deposited in the SSBD repository (https://doi.org/10.24631/ssbd.repos.2022.10.256). Supplemental material available at GENETICS online.
