# Supplementary material for: Osiris gene family defines the cuticle nanopatterns of Drosophila
Source: Genetics. 2024 Apr 23;227(2):iyae065. doi: 10.1093/genetics/iyae065 (PMC11151929; doi:10.1093/genetics/iyae065)
Supplement: iyae065_Supplementary_Data [file iyae065_supplementary_data.zip › Legend_for_Supplemental_Materials_GENETICS-2024-306978.docx]

# **Legend for Supplemental Materials**

## **Supplemental Table**

### **Table S1. Key Resource Table**

Reagent, Drosophila strains, equipment, and software used in this study.

### **Table S2. Effect of transgenic RNAi experiments**

List of UAS RNAi strains targeting *Osiris* genes collected from the National Institute of Genetics, Bloomington Stock Center, and Vienna Drosophila Stock Center. Their effects on cell viability when crossed with da-Gal4, actin-Gal4, or neur-Gal4 are shown.

**Table S3. List of *Osiris* knockout strains and guide RNA sequences**

**Sheet “List of Osiris mutants”**

List of mutations for each *Osi* genes.

**Sheet “KO fly series”.**

List of sequenced *Osi* gene alleles. The shaded row of each gene marked as WT shows the targeted wild-type sequence. Sequences corresponding to the guide RNA are underlined and the PAM sequences are in bold. Multiple guide RNAs have been designed for *Osi1, Osi6, and Osi22*. Only the mutants with out-of-frame in/del mutations were saved for further analysis.

**Sheet “gRNA oligo&vector”.**

List of oligonucleotide sequences used to build guide RNA vectors.

## **Supplementary Figures**

### **Figure S1. Co-staining with anti-phosphotyrosine and anti-Futsch antibodies identifies trichogen and tormogen cells.**

Enlarged views of mechanosensory organs in An3. The anti-Futsch (22C10) antibody (yellow) strongly labeled the shaft of the trichogen and weakly labeled the cytoplasm of the soma. The phosphotyrosine (pY) antibody (green) labels the cell outline. These markers allowed the identification of *Osi11* expression in trichogens and *Osi12* in tormogens, and were used throughout this study.

### **Figure S2. mRNA FISH patterns of nine *Osi* genes in developing pupal head (42 hours APF).**

Representative images of nine *Osi* gene expression patterns that were judged to be undetectable. Some magenta signals were nonspecific reactions to the pupal cuticle remnants. Tissue outlines were marked with DAPI staining (cyan).

### **Figure S3. Additional expression patterns of *Osi* genes.**

This Figure is separated in five sheets (S3-1 to S3-5). Red, FISH signals of *Osi* RNAs; green, anti-phosphotyrosine; yellow, anti-Futsch (22C10) staining; cyan, DAPI.

**S3-1**

***Osiris1.*** Expression was detected in the basal cylinder and distal cells of the Arista. In the eye, it was detected in primary pigment cells and in unidentified cells below the photoreceptor cells in the ommatidia.

***Osiris3.*** Its expression is broadly detected in the epidermis. It is also expressed in the tormogens Mp, Lab, and An3.

***Osiris4.*** Two types of expression were observed in the pupal heads sampled at 42 h APF. In the whole-head view, the example on the left shows the expression in the eye, mechanosensory organ, and gustatory organ (the same as that shown in Figure 1). The second example shows expression in the pseudotrachea of the labella. High-magnification views of Mp showed expression in mechanosensory trichogens only in one case and in both trichogens and tormogens in another case. The example in the Lab shows an expression in the gustatory tormogen. In An2, tormogen expression was high, but trichogen expression was weak. In the eye, the expression of primary pigment cells, tormogen, and trichogen cells of the interommatidial mechanosensory bristles was observed.

**S3-1**

***Osiris5.*** This gene was specifically expressed in An3 (middle: projection of the anterior surface, right: single slice) in a pattern enriched in the bottom-lateral territory. *Osi5* was not detected in the Mp.

***Osiris6.*** Expression was detected in cells adjacent to the pseudotrachea in the Lab (middle), and in primary pigment cells in the compound eye.

***Osiris7.*** Broad expression was detected in epidermal cells. *Osi7* was also expressed in tormogen cells of mechanosensory organs of An2 (top middle: projection view, top right: single slice), eye (interommatidial bristle cells: lower 5.4 µm deep slice) and Mp (lower right). It was also expressed in the primary pigment cells and cone cells of the compound eye (lower left, Figure 4) and in the pseudotrachea cells of Lab.

**S3-3**

***Osiris8.*** Expression in trichogen cells of the mechanosensory and gustatory organs. The signals in the eye are the trichogen cells of the interommatidial mechanosensory cells. In the Arista, *Osi8* is expressed on the dorsal side of the basal cylinder and distal cells. In the Mp, the trichogen cells of the mechanosensory organ express *Osi8*.

***Osiris9.*** Expressed in the epidermis (whole head, An2, An3, Mp), primary pigment cells, and unidentified cells (7.56 µm deep section) of the eye. In the Lab, *Osi9* expression in the epidermal cells was low or absent.

***Osiris11.*** Expression in trichogen cells of mechanosensory and gustatory organs. It is also expressed in the Arista.

**S3-4**

***Osiris12.*** It is expressed in tormogen cells of mechanosensory (Eye, Mp, An2), gustatory (Lab), and olfactory (An2) organs. It is also expressed in a subset of the epidermis of Lab (medial sections), An3, and Arista cells, forming a distal ring of the basal cylinder.

***Osiris13.*** Expression in trichogen cells of the olfactory organs in An3 and Mp. These are the sb of An3 and Mp. The expression in An3 was the st and sc.

***Osiris16.*** expression in a small subset of the trichogen cells of An3, but not in Mp. This expression was likely to occur in the sensilla coeloconica (sc).

**S3-5**

***Osiris21.*** Expression in trichogen cells of all mechanosensory organs (examples in An2 and Mp). It was also expressed in the gustatory organs (Figure 3).

***Osiris22.*** Broadly expressed in the epidermis. Examples of An2 and An3 (two focal planes), Mp, and Lab, are presented. At the Arista, *Osi22* was expressed in the dorsal half of the basal cylinder.

***Osiris23* (*gore-tex*)*.*** Sb expression in An3 and Mp trichogen cells Weaker expression was detected in the bottom-medial part, likely corresponding to st (Figure 6).

***Osiris24.*** Expression in trichogen cells of mechanosensory (An2) and olfactory (An3, Mp) organs. The weak signals in the epidermis were nonspecific.

### **Figure S4. Co-labeling of *Osi13* (red) and *Osi23* (green) in maxillary palp.**

The expression of these two genes mostly overlapped with some differences (cell b in the middle panel).

### **Figure S5. *Osi17* KD and KO phenotypes in adult and third instar imaginal discs.**

1. Wing expansion defects in *Osi17* RNAi (VDRC37457) are induced by actin-Gal4. Treated animals also exhibited a kinked leg phenotype (enlarged view with an asterisk).
2. Wing-expansion defects in animals with homozygous *Osi17 knock*-*out* mutants induced by wing pouch-specific Ubx-FLP recombination.
3. Expression of *Osi17* RNAi in the trachea did not cause wing defect.
4. Wing, leg, and haltere imaginal discs of third-instar larvae. GFP markers for wild-type (green) and F-actin (phalloidin, red). Mosaicism is induced in the pouch regions of the wing and halter discs. No mosaicism was detected in the notum, trachea (tr), air sac primordium (as), or adult muscle precursor (amp).

## **Supplementary Movies**

### **Movie S1. Serial cross-sectional views of *Osi23* expression in An3.**

A confocal stack of An3 labeled *Osi23*/*gox* RNA (magenta) was computationally flattened, separated into lateral (top) and medial (bottom) halves, and presented as a series of sections moving from the surface to the interior.
