# Supplementary material for: Osiris gene family defines the cuticle nanopatterns of Drosophila
Source: Genetics. 2024 Apr 23;227(2):iyae065. doi: 10.1093/genetics/iyae065 (PMC11151929; doi:10.1093/genetics/iyae065)
Supplement: iyae065_Supplementary_Data [file iyae065_supplementary_data.zip › Supplemental_Table_S1_GENETICS-2024-306978.docx]

**Supplementary Table S1.**

**Key resource table**

| REAGENT or RESOURCE | SOURCE | IDENTIFIER |
| --- | --- | --- |
| Antibodies | | |
| P-Tyr-1000 MultiMab Rabbit mAb mix | Cell Signaling Technology | Cat# 8954, RRID:AB_2687925 |
| futsch antibody | DSHB | Cat# 22C10, RRID:AB_528403 |
| Goat anti-Rabbit IgG, Alexa Fluor 488 | Thermo Fisher Scientific | Cat# A-11034, RRID:AB_2576217 |
| Goat anti-Mouse IgG, Alexa Fluor 633 | Thermo Fisher Scientific | Cat# A-21052, RRID:AB_2535719 |
| Anti-Digoxigenin-POD, Fab fragments | Roche | 11207733910 |
| Streptavidin-POD | Roche | 11089153001 |
| Chemicals, Peptides, and Recombinant Proteins | | |
| Alexa Fluor 568 Phalloidin | Thermo Fisher Scientific | Cat# A12380 |
| TSA Fluorescein | PerkinElmer | SAT701001KT |
| TSA Cyanine 3 | PerkinElmer | SAT704A001KT |
| Paraformaldehyde | TAAB | Cat# P001 |
| Glutaraldehyde EM Grade | TAAB | Cat# G011/1 |
| 16% Formaldehyde Solution (w/v) | Thermo Scientific | Cat# 28908 |
| Blocking Reagent | Roche | Cat# 11 096 176 001 |
| DIG RNA Labeling Mix, 10x conc. | Roche | Cat. No. 11 277 073 910 |
| Biotin RNA Labeling Mix, 10x conc. | Roche | Cat. No. 11 685 597 910 |
| Antifade Mounting Medium with DAPI | Vector Laboratories | H-1200 |
| Xylene | Wako | 244-00086 |
| Acetone | Wako | 016-00346 |
| Ethanol | Wako | 057-00451 |
| PBS (10×) | nacalai tesque | Cat# 27575-31 |
| Molecular Sieves 3A 1 / 16 | nacalai tesque | Cat# 04170-15 |
| Experimental Models: Organisms/Strains | | |
| *D. melanogaster: Oregon R* |  | N/A |
| *D. melanogaster: neur-Gal4* | (Bellaïche *et al.* 2001) | FBti0017282 |
| *D. melanogaster: act-Gal4* | Direct fusion of Gal4 to Act5C promoter | N/A |
| *D. melanogaster: da-Gal4* | (Wodarz *et al.* 1995) |  |
| *D. melanogaster: btl-Gal4* | (Shiga *et al.* 1996) | DGRC 109128 |
| *D. melanogaster: y[1] w{*}* | Akira Nakamura |  |
| *D. melanogaster: y[1] w[*] P{w[+mC]=Ubx-FLP}1* | Jurgen Knoblich | RRID:BDSC_42718 |
| *D. melanogaster: w[1118]; P{ry[+t7.2]=neoFRT}82B P{w[+mC]=Ubi-GFP(S65T)nls}3R P{ry[+t7.2]=A92}RpS3[Plac92]/TM6C, Sb[1]* | Bloomington *Drosophila* Stock Center | RRID:BDSC_5627 |
| *D. melanogaster: RNAi strains* | See supplementary Table S1 |  |
| *D. melanogaster: Osi knockout strains* | See supplementary Table S1 | This study |
| Microscope | | |
| Confocal microscope | Olympus | FV1000 |
| FE-SEM | JEOL | JSM-IT700HR |
| Software and Algorithms | | |
| ImageJ-Fiji | LOCI | https://fiji.sc |
