# Supplementary material for: Osiris gene family defines the cuticle nanopatterns of Drosophila
Source: Genetics. 2024 Apr 23;227(2):iyae065. doi: 10.1093/genetics/iyae065 (PMC11151929; doi:10.1093/genetics/iyae065)
Supplement: iyae065_Supplementary_Data [file iyae065_supplementary_data.zip › Tables_1_GENETICS-2024-306978.docx]

**Table 1. *Osi* expression patterns.**

|  | Trichogen |  |  | Tormogen | lens | epi | pseudotrachea | arista | phenotype |
| --- | --- | --- | --- | --- | --- | --- | --- | --- | --- |
| *Osi* | olf | mech | gustatory |  |  |  |  |  |  |
| 1 |  |  |  |  | + |  |  | + |  |
| 3 |  |  |  | + |  | + |  |  |  |
| 4 | + | + | + | + | + |  | + | + | cn |
| 5 | + |  |  |  |  |  |  |  |  |
| 6 |  |  |  |  | + |  | + |  |  |
| 7 |  |  |  | + | + | + | + |  |  |
| 8 |  | + | + |  |  |  |  | + |  |
| 9 |  |  |  |  | + | + |  |  | cn |
| 11 |  | + | + |  |  |  |  | + | gb |
| 12 |  |  |  | + |  | + |  | + |  |
| 13 | + |  |  |  |  |  |  |  |  |
| 16 | + |  |  |  |  |  |  |  |  |
| 21 |  | + | + |  |  |  |  |  |  |
| 22 |  |  |  |  |  | + |  | + |  |
| 23 | + |  |  |  |  |  |  |  | sb, tr |
| 24 | + | + |  |  |  |  |  |  |  |

Abbreviations: cn, corneal nipple; gb, gustatory bristle; sb, sensilla basiconica; tr, sensilla trichordia.
