# Supplementary figures and images for: Osiris gene family defines the cuticle nanopatterns of Drosophila
Source: Genetics. 2024 Apr 23;227(2):iyae065. doi: 10.1093/genetics/iyae065 (PMC11151929; doi:10.1093/genetics/iyae065)

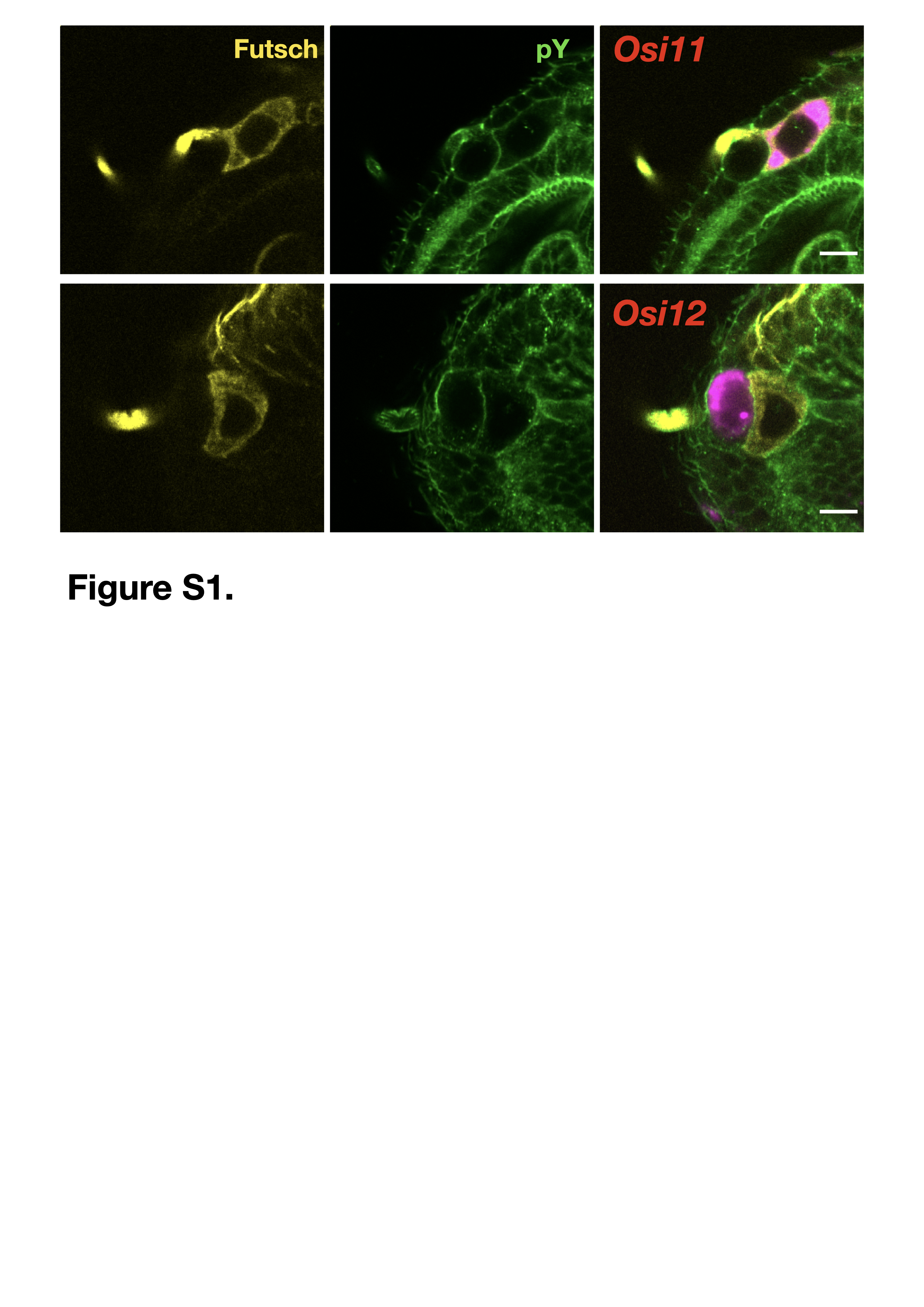

Supplement: iyae065_Supplementary_Data [file iyae065_supplementary_data.zip › Supplemental_Figure_S1_GENETICS-2024-306978.tif]

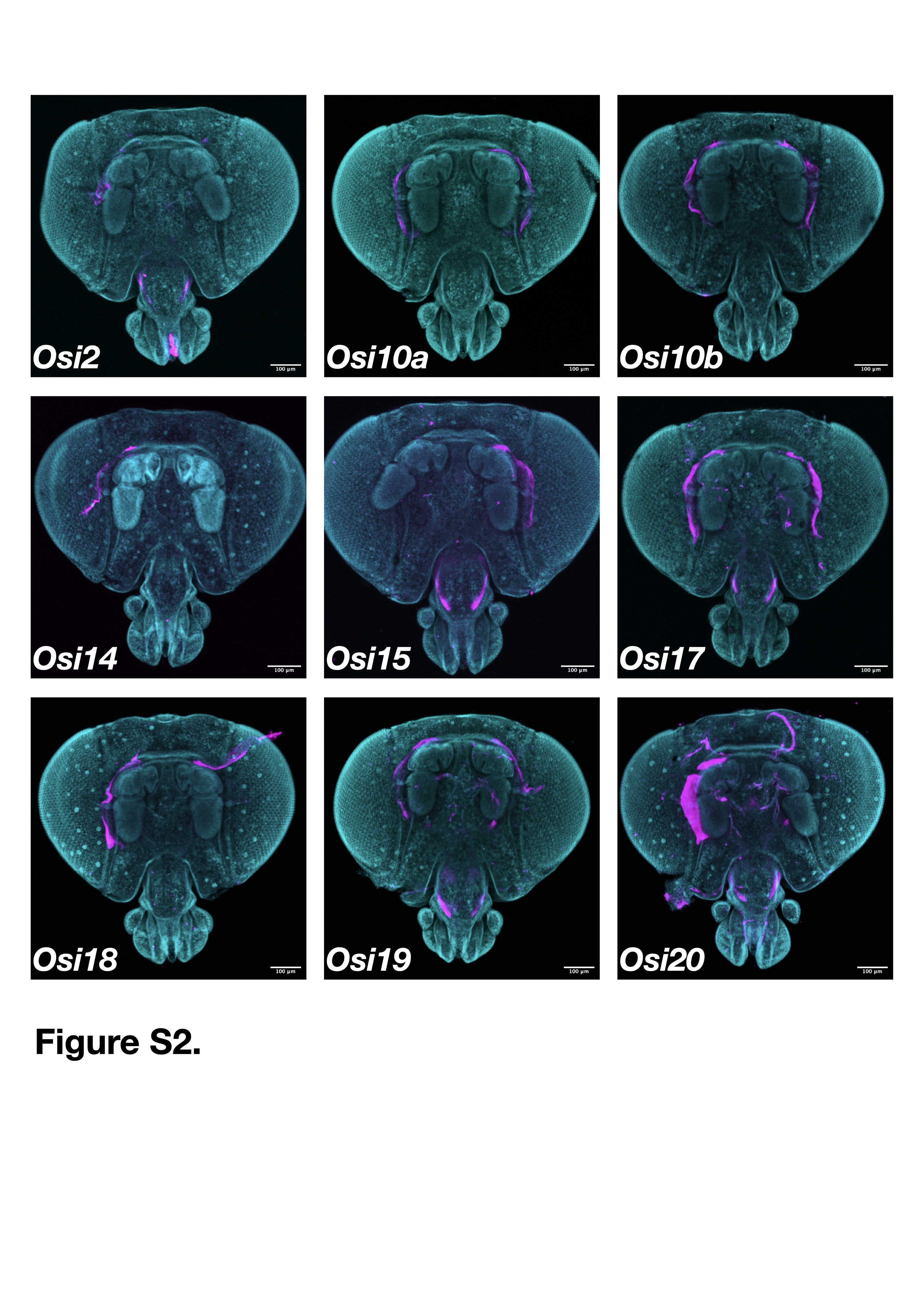

Supplement: iyae065_Supplementary_Data [file iyae065_supplementary_data.zip › Supplemental_Figure_S2_GENETICS-2024-306978.tif]

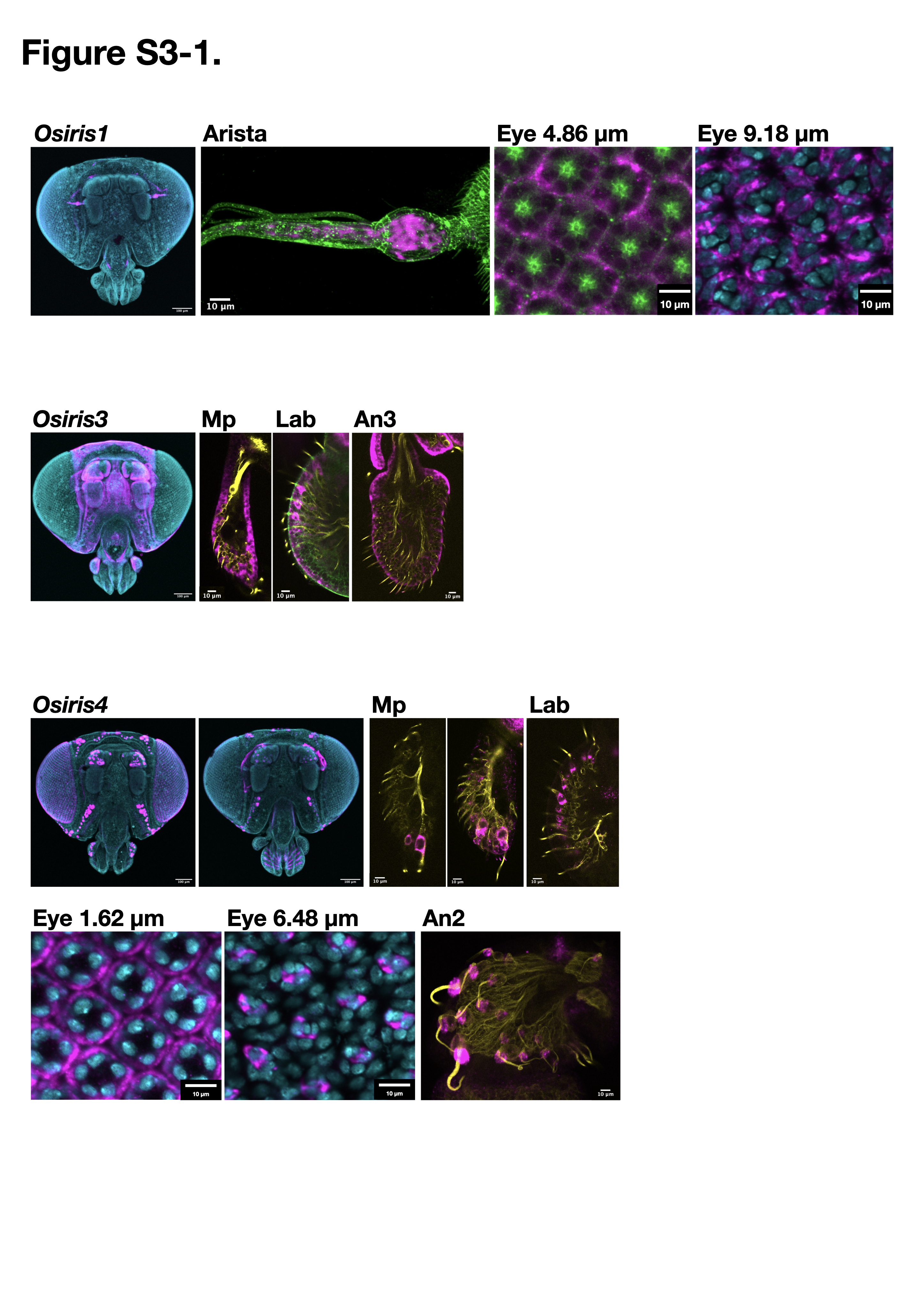

Supplement: iyae065_Supplementary_Data [file iyae065_supplementary_data.zip › Supplemental_Figure_S3-1_GENETICS-2024-306978.tif]

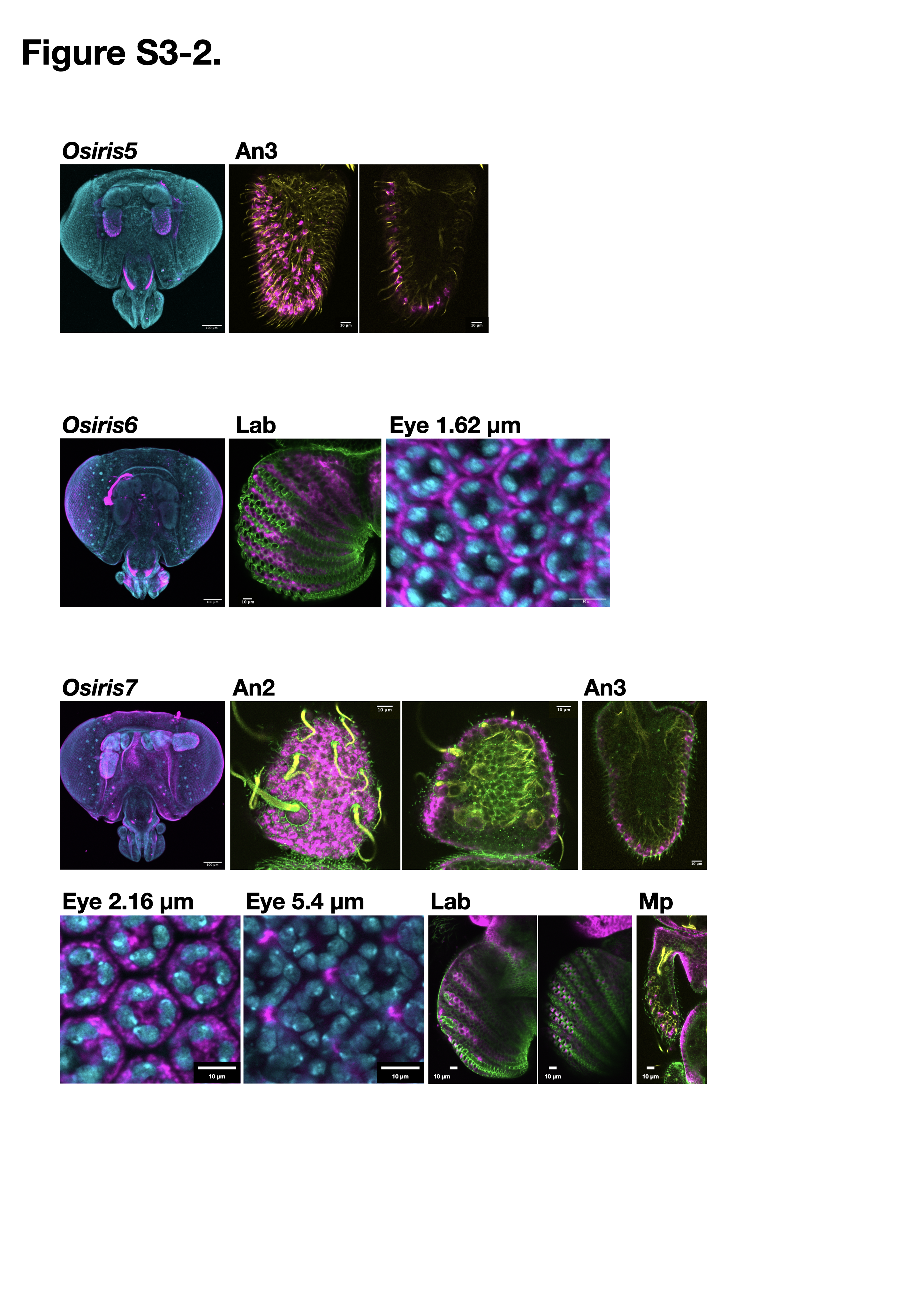

Supplement: iyae065_Supplementary_Data [file iyae065_supplementary_data.zip › Supplemental_Figure_S3-2_GENETICS-2024-306978.tif]

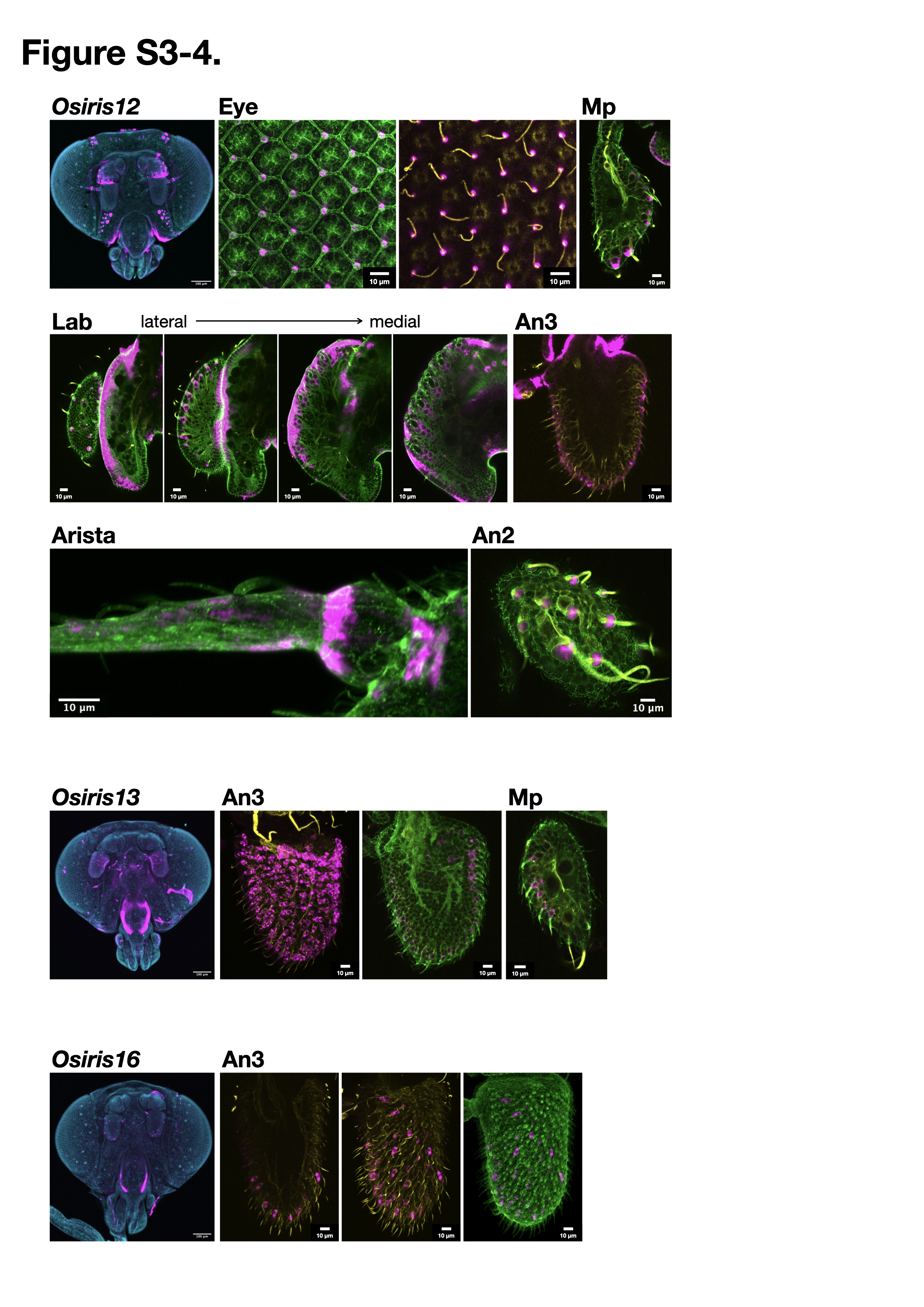

Supplement: iyae065_Supplementary_Data [file iyae065_supplementary_data.zip › Supplemental_Figure_S3-4_GENETICS-2024-306978.tif]

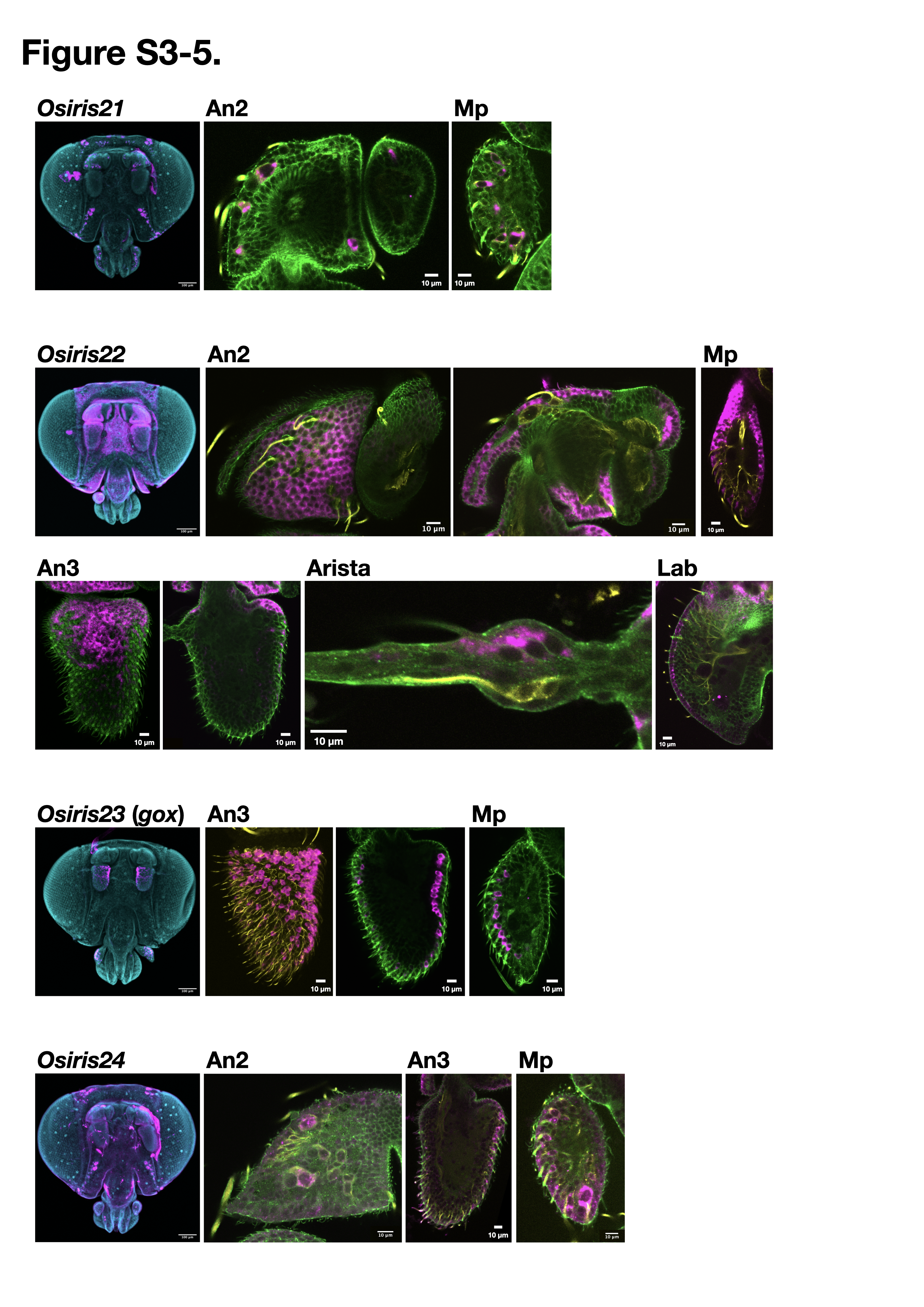

Supplement: iyae065_Supplementary_Data [file iyae065_supplementary_data.zip › Supplemental_Figure_S3-5_GENETICS-2024-306978.tif]

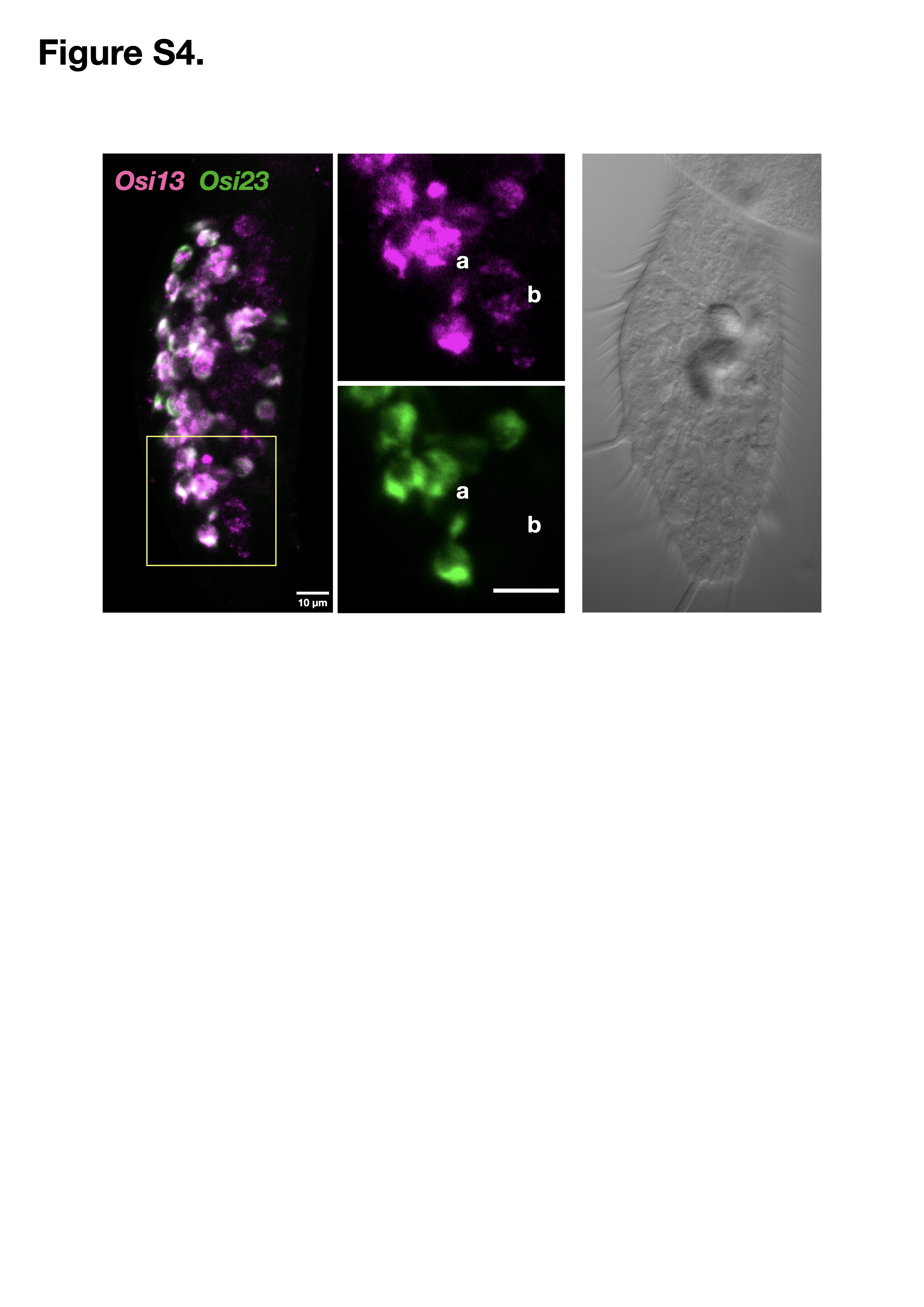

Supplement: iyae065_Supplementary_Data [file iyae065_supplementary_data.zip › Supplemental_Figure_S4_GENETICS-2024-306978.tif]

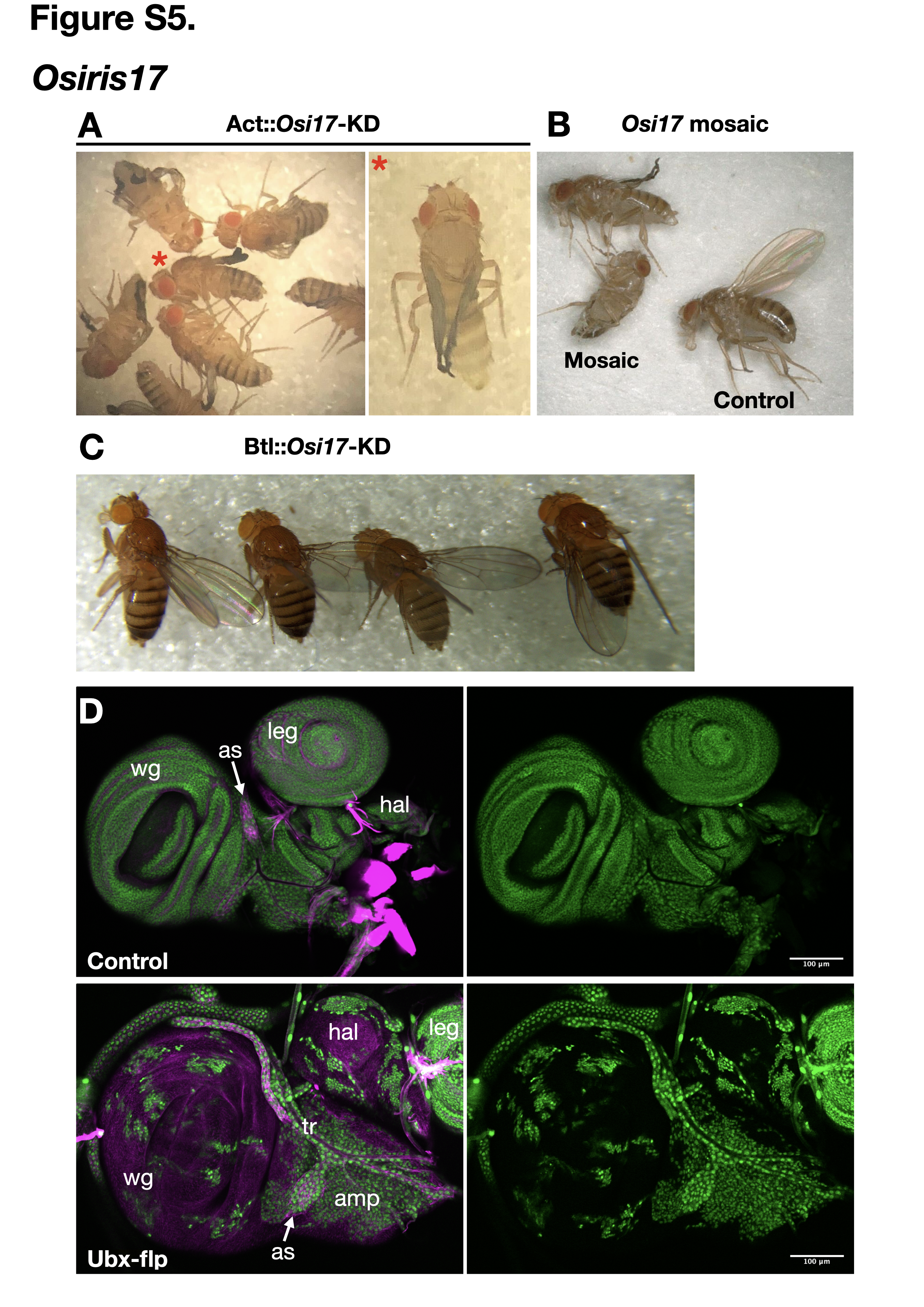

Supplement: iyae065_Supplementary_Data [file iyae065_supplementary_data.zip › Supplemental_Fubure_S5_GENETICS-2024-306978.tif]

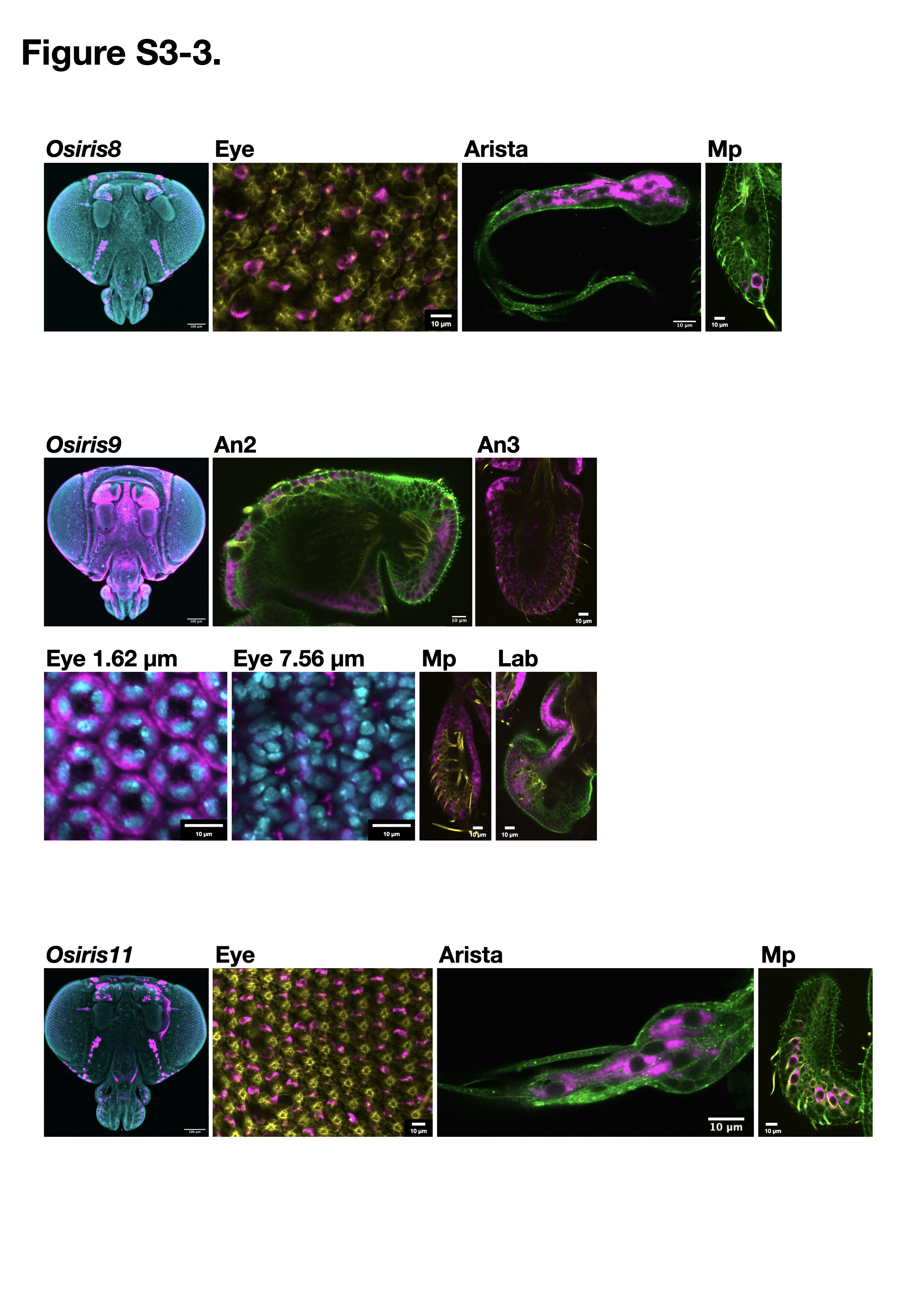

Supplement: iyae065_Supplementary_Data [file iyae065_supplementary_data.zip › Supplemental_Fugure_S3-3_GENETICS-2024-306978.tif]
